# Fetal Lead Exposure and Infant Mental Development Index

**DOI:** 10.1289/ehp.115-a186a

**Published:** 2007-04

**Authors:** Roberto Ronchetti

**Affiliations:** Clinica Pediatrica, Ospedale Sant’Andrea, Rome, Italy, E-mail: roberto.ronchetti@ospedalesantandrea.it

All of the authors of Ronchetti et al. (2006) read with great interest the article of Hu et al. (2006); their excellent experimental data fully supports the hypotheses and conclusions we reported in an our recent literature review on lead neurotoxicity in children (Ronchetti et al. 2006). Hu et al. (2006) show that in predicting the loss of mental development index (MDI) in 2-year-old children, the plasma lead level is better than the whole blood lead level, and measurements made in the first trimester of pregnancy are better than measures obtained later in pregnancy or in cord blood.

One issue that remains unexplained, however, is the enormous scatter of the individual points around the correlation line between plasma lead concentrations and the MDI. Equally wide scatter also emerges in the similar figure reported by Canfield et al. (2003). This finding suggests that plasma lead concentrations (or whole blood lead levels used by Canfield et al.) are not the direct determinants of lead neurotoxicity and that the relationship between lead concentrations measured in blood and the decrease in MDI are significantly influenced by other biological factors (Mushak 1998).

Lead is dissolved in a circular river (blood circulation); every day the river is contaminated by a relatively small affluent (daily lead intake) and purified by some effluents (excretion). however, it is in contact and is heavily influenced by a large lake (long-term bone stores), which can be heavily contaminated. The lead contamination in the lake slowly but continuously influences the lead concentration in the river, and thereby tends to contaminate all the other small lakes (body tissues and organs including brain) with which the river comes in contact. As long as we continue to measure the lead concentration in the river, we will have a proxy variable to define the real situation in the body and in the brain.

This scenario engenders concepts that are important in understanding and preventing lead neurotoxicity. First, when women born after lead was removed from gasoline become mothers, they will be persons whose “big lake” is less contaminated. Second, as Hu et al. (2006) stated, even at the present time we have the means (e.g., calcium supplementation from the beginning of the pregnancy) to close some of the gates between the big lake and the river (we can at least partially avoid maternal bone lead mobilization during pregnancy).

From a scientific point of view, we could understand lead toxicology far better and also plan more effective preventive interventions if we include the measure of bone lead concentrations in mothers and children in epidemiologic studies.
